# Microwave-assisted synthesis of water-dispersed CdTe/CdSe core/shell type II quantum dots

**DOI:** 10.1186/1556-276X-6-399

**Published:** 2011-05-27

**Authors:** Li-Man Sai, Xiang Yang Kong

**Affiliations:** 1 School of Material Science and Engineering, Shanghai Jiao Tong University, Dongchuan Road 800, Shanghai 200240, People's Republic of China

## Abstract

A facile synthesis of mercaptanacid-capped CdTe/CdSe (core/shell) type II quantum dots in aqueous solution by means of a microwave-assisted approach is reported. The results of X-ray diffraction and high-resolution transmission electron microscopy revealed that the as-prepared CdTe/CdSe quantum dots had a core/shell structure with high crystallinity. The core/shell quantum dots exhibit tunable fluorescence emissions by controlling the thickness of the CdSe shell. The photoluminescent properties were dramatically improved through UV-illuminated treatment, and the time-resolved fluorescence spectra showed that there is a gradual increase of decay lifetime with the thickness of CdSe shell.

## Introduction

Semiconducting nanocrystals such as quantum dots (QDs) have attracted more attention due to their unique optical properties and many potential applications including nanolasers, biolabelings, and photovoltaics, etc. [1,2]. The unique optical properties are featured as narrow emission spectra, continuous absorption band, high chemical and photobleaching stability, and surface functionality. To date, QDs have various nanostructured configurations, typically as core/shell heterostructure QDs, where two different semiconductors are incorporated into a single colloidal QD [3]. There are type I and type II core/shell QDs with different carrier localizations, depending on the band structure offsets between the semiconducting core and the shell [4]. Type I is where both the electrons and holes are confined in the core, in contrast, type II is where the electrons and holes are separated between the core and the shell, giving rise to a significant increase in the exciton lifetime with possible applications in photovoltaics [5]. Specifically, it has been reported that the CdTe/CdSe core/shell QDs exhibit type II band alignment facilitating charge separations upon absorption of visible light for solar cells [6].

Recently, the high-quality CdTe/CdSe heterostructure QDs have been successfully synthesized via colloidal chemical routes [7-9]. However, these synthetic methods cost several hours in an organic solvent at high temperature, and the product easily performed the agglomeration with broad size distribution. It is desirable to develop a facile method for fast synthesis of highly fluorescent type II core/shell QDs in aqueous ion solution. A microwave-assisted synthesis is an attractive method employed routinely for the synthesis of nanocrystals due to the advantages of the reaction selectivity and high efficiency for obtaining the controllable products [10-12]. In this paper, we employed the microwave-assisted synthesis in aqueous solution for the water-dispersed CdTe/CdSe core/shell type II nanocrystals. The optical properties of as-prepared CdTe/CdSe nanocrystals can be optimized in the presence of Cd^2+ ^and mercaptopropionic acid by UV-illuminated treatment. The photoluminescence quantum yield (PLQY) of the as-prepared QDs was enhanced from 12% to as high as 45%. These aqueous-dispersed CdTe/CdSe core/shell type II QDs may have potential applications in solar cells.

## Experimental methods

### Microwave-assisted synthesis of CdTe core QDs

For monodispersed CdTe core QD synthesis, the CdTe precursor solution was prepared by adding a freshly prepared NaHTe solution to a N_2_-saturated CdCl_2 _solution in the presence of the stabilizer of 3-mercaptopropionic acid (MPA). The molar ratio of Cd^2+^/MPA/HTe^- ^was set as 1:2.5:0.2. The as-prepared CdTe precursor solution is subjected to microwave irradiation for about 2 min at 100°C, and is naturally cooled down lower than 50°C. The CdTe core QDs stabilized with MPA were concentrated from the solution and were precipitated with 2-propanol by centrifugation, and then re-dissolved in ultrapure water.

### Microwave-assisted synthesis of CdTe/CdSe core/shell QDs

The CdTe/CdSe precursor solution was prepared by adding a certain amount of postprepared CdTe core QDs to a N_2_-saturated solution mixed with CdCl_2_, NaHSe, and MPA in pH 11.2. The concentration of Cd^2+ ^was fixed at 1.25 mM. The high-quality CdTe/CdSe QDs were prepared in a very short time, and the sizes of the QDs were controlled on the basis of regulating the reaction time of microwave irradiation. The samples were taken when the temperature decreased naturally to lower than 50°C and centrifuged for high concentration.

### Structural characterizations and spectroscopic measurements

The as-prepared QD samples were precipitated by 2-propanol and dried in a vacuum oven for X-ray diffraction (XRD) characterization. The XRD patterns were recorded from a Rigaku D/max-γB diffractometer. The samples for transmission electron microscopy (TEM) were prepared by dropping the aqueous CdTe/CdSe solution onto carbon-coated copper grids with the excess solvent evaporated. The TEM images were recorded from a JEOL JEM 2100 electron microscope (JEOL, Tokyo, Japan) operated at 200 kV.

The UV-Vis absorption spectra were recorded with a Shimadzu UV-3150 UV-Vis-near-infrared spectrophotometer (Shimadzu Corporation, Columbia, MD, USA)The photoluminescence (PL) measurements were performed using a Shimadzu RF-6301PC spectrofluorimeter (Shimadzu Corporation, Columbia, MD, USA). The PLQY of QDs at room temperature was estimated using standard method [13]. The optical density at the excitation wavelength of the Rhodamine 6G (R6G) and the QD samples in the solution were set to a similar value. The wavelength of the excitonic absorption peak of the QDs was set as the excitation wavelength for measurement. The integrated PL intensities of the QD and R6G were calculated from the fully corrected fluorescence spectrum. The PLQY of the QD samples was finally obtained by comparing the integrated PL intensities of the QDs and R6G. The UV-illuminated treatment of the samples was done with three UV lamps, and the intensity of the illuminated light was 16 W.

## Results and discussion

**Figure 1:**
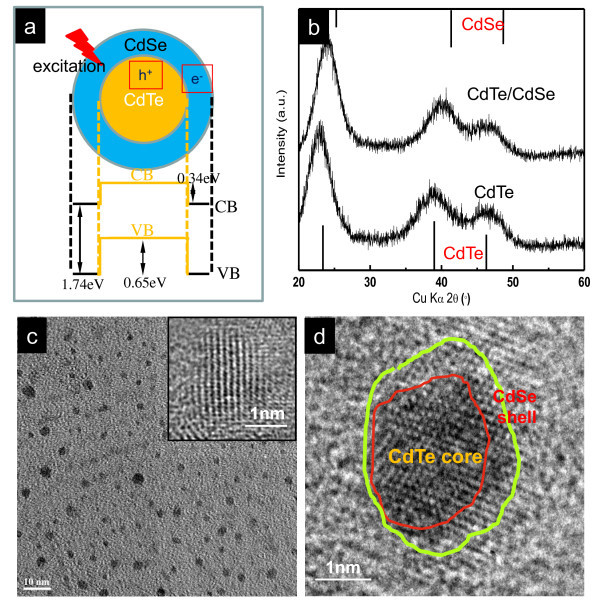
**Band structure alignment**. **(a) **Alignment of the conduction and valence band edges for CdTe/CdSe core/shell type II heterostructures. **(b) **XRD patterns of CdTe and CdTe/CdSe core/shell QDs via microwave-assisted synthesis. The black bars at the bottom represent the XRD pattern of bulk CdTe (cubic). The black bars at the top represent the XRD pattern of bulk CdSe (cubic). **(c) **TEM bright field image of the CdTe core QDs in low magnification, the inset for the HRTEM image of CdTe QD with cubic structure. **(d) **HRTEM image of CdTe/CdSe core/shell QD, different contrasts corresponding to the CdTe core and CdSe shell, respectively. The thickness of the shell is about four to five atomic layers.

The typical band structure alignment of the conduction and valence band edges for the CdTe/CdSe core/shell type II heterostructures is shown in Figure 1a. With regard to the core/shell structures, the separation of the hole and the electron can be achieved upon excitation. The hole is mostly confined in the CdTe core while the electron is in the CdSe shell. The as-received CdTe and CdTe/CdSe core/shell QDs via microwave-assisted synthesis were examined systematically from the crystallography structure to the unique optical properties.

Figure 1b shows the XRD patterns of the original CdTe cores and the corresponding CdTe/CdSe QDs. The diffraction pattern of the bare CdTe cores is consistent with that of the bulk cubic CdTe structure, represented by the diffraction peaks at 23.5°, 39.1°, and 46.5°. Such broad diffractive peaks are typical for the crystals with nanoscale size. The diffraction pattern of CdTe/CdSe QDs moves gradually toward a higher angle. These pattern configurations indicate clearly the formation of heterostructure of CdTe/CdSe QDs. Similar results in the diffraction patterns are consistent with previous reports as well [9]. In addition, the pattern of peak widths and shapes is nearly unchanged, which indicates it should be the CdS/CdTe core/shell structure rather than the CdTe_x_S_1-x _alloyed structure.

TEM images of the as-prepared CdTe and CdTe/CdSe QDs are shown in Figure 1c,d, respectively. From the low magnification image, the CdTe core QDs appear as the size of about 2 nm spherical particles with good monodispersity. The existence of a well-resolved lattice fringe on the HRTEM image further confirms the crystalline structure of CdTe. Figure 1d illustrates a typical CdTe/CdSe core/shell QD from different contrasts corresponding to a CdTe core and CdSe shell, respectively. The thickness of a CdSe shell is about four to five atomic layers.

**Figure 2:**
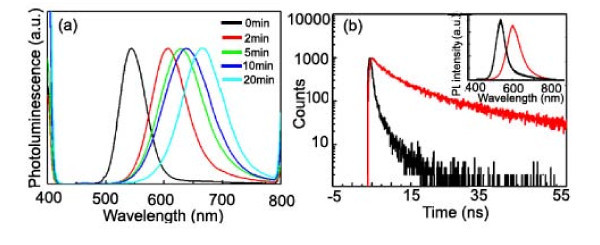
**Optical performance of CdTe cores and corresponding CdTe/CdSe type II QDs by means of microwave-assisted synthesis.** The optical performance of a series of original CdTe cores and corresponding CdTe/CdSe type II QDs by means of microwave-assisted synthesis. The QD samples are excited at their first excitation absorption peak, and The PL intensities of all the samples are normalized. **(a) **Fluorescence spectra of CdTe cores (black line) and CdTe/CdSe core/shell QDs with controlled reaction time under microwave irradiation, giving rise to the different CdSe shell thickness surrounding the CdTe core. The molar ratio of [Cd^2+^]/[MPA]/[Se^2-^] was set as 1:2.4:1, the concentration of Cd^2+ ^was fixed at 1.25 mM, reaction temperature at about 100°C, and reaction time range from 2 min to approximately 20 min. **(b) **Fluorescence decay curves of a CdTe core (black line) and the CdTe/CdSe core/shell QDs (red line). The lifetimes were recorded at the maxima of the emission with the excitation wavelength of 371 nm. The inset picture indicates the fluorescence spectra of the corresponding QDs. The decay lifetime increases dramatically due to the coating of CdSe shell (reaction time of about 5 min under microwave irradiation).

The optical performance of a series of original CdTe cores and corresponding CdTe/CdSe type II QDs synthesized at 100°C with different reaction times were examined, as shown in Figure 2a. It shows the continuous red shift in emission with the coating of CdSe shells onto CdTe core QDs. When the reaction or coating CdSe shell time is up to 20 min, the emission wavelength shifted to 665 nm, with an increase of 120 nm compared to the CdTe core QDs whose emission peak appeared at 545 nm. The thickness of the CdSe shell is observed as up to four or five atomic layers surrounding the CdTe cores with a 2-nm radius. As the reaction time increases, the CdSe shell coating proceeds and results in longer wavelengths of the emission shift. This clearly indicates the strong type II characteristics as the excitons become more spatially separated by thicker shells. Therefore, the PL peak can be assigned to an indirect excitation, originating from the radiative recombination of electron hole pairs across the core/shell interface [14,15].

Lifetime measurements allow us to probe the degree of wavelength overlap of the carriers, or the oscillator strength of the transition. Figure 2b shows a PL decay of a CdTe/CdSe QD sample dispersed in water. The CdTe/CdSe QD sample with the thickest shell (about five atomic layers) was selected for the PL decay measurement. The CdTe and CdTe/CdSe QD solutions are diluted to achieve the same absorbance value (0.1) at the wavelength of their first excitation absorption peak. As shown in Figure 2b, an increase of the decay lifetime is observed with the increase of the CdSe shell. It was reported that the wave function overlap integral is inversely proportional to the radiative lifetime [16], giving rise to the charge separation of the electron and hole for longer lifetime values. This undoubtedly reveals the type II characteristics of our CdTe/CdSe QDs demonstrating the spatially separated excitons.

**Figure 3:**
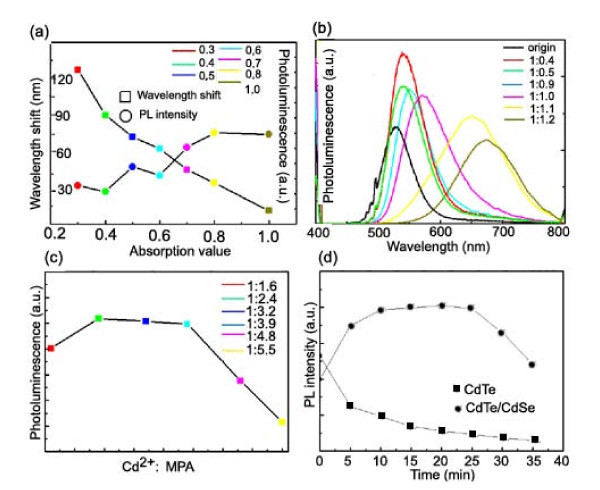
**Wavelength shift and PL intensity of CdTe/CdSe core/shell QDs**. The PL intensities of the as-prepared QDs solutions were estimated from the samples diluted to a certain concentration with the same absorbance values at the wavelength of their first excitation absorption peak. **(a) **Wavelength shift and corresponding PL intensity of CdTe/CdSe QDs versus different absorption values of the CdSe precursor solution. **(b) **PL spectra of CdTe/CdSe core/shell QDs synthesized with different [Se^2-^]/[Cd^2+^] molar ratio. **(c) **PL intensity of CdTe/CdSe QDs versus different [Cd^2+^]/[MPA] molar ratio. **(d) **PL intensity of CdTe and CdTe/CdSe QDs versus the UV-illuminated treatment time.

In order to obtain the appreciated properties of the as-received core/shell QDs, the synthesis conditions were optimized. Figure 3a shows that the wavelength shift and PL intensity of CdTe/CdSe QDs are strongly influenced by the CdTe concentration. All the CdTe/CdSe QD solutions were diluted to a certain concentration for the same absorbance values at the wavelength of their first excitation absorption peak, which was also used for the excitation of the QD samples in the PL measurement. The molar ratio of [Cd^2+^]/[MPA]/[HSe^-^] was set as 1:2.4:1, the concentration of Cd^2+ ^was fixed at 1.25 mM, the reaction temperature was set at 100°C, and the reaction time was about 3 min. Here, we use the absorption values at the first excitation absorption peak of the CdTe QDs to represent the concentration of CdTe core QDs dispersed in the CdSe precursor solution. The wavelength shift refers to the emission wavelength difference of CdTe/CdSe core/shell QDs and CdTe core QDs. When this concentration was small, there would be much Se^2- ^existing around each CdTe QD, therefore resulting in the fast coating of the CdSe shell. The wavelength shift increased with the thicker CdSe shell. When the concentration was too high (e.g., absorption value at 1.0), there would be too few Se^2- ^for shell coating, and the emission wavelength almost did not shift. On the other hand, the PL intensity gradually decreased with the decreasing absorption value, which shows that fast coating will lead to a larger amount of surface defects and thus decrease of PL intensity. In our experiment, the proper range of CdTe absorption value was set about 0.5 to 0.7.

The PL spectra of CdTe/CdSe core/shell QDs synthesized with different [Cd^2+^]/[Se^2-^] molar ratio is shown in Figure 3b. The absorption values of the CdTe core were fixed at 0.6. The molar ratio of Cd^2+^/MPA was set as 1:2.4, and the reaction temperature and time were about 100°C and 3 min, respectively. It is shown that the emission peak of CdTe/CdSe QDs dramatically shifted to longer wavelength with the increase amount of Se^2-^. The larger amount of Se^2- ^existing in the solution will give rise to the faster coating of the CdSe shell as well as more surface defects. The [Cd^2+^]/[Se^2-^] molar ratio is optimized around 1:1 for good optical performance.

It is also found that the MPA stabilizer plays an important role on the optical performance of the CdTe/CdSe QDs. The molar ratio of [Cd^2+^]/[Se^2-^] was set as 1:1, the absorption value of the CdTe core was fixed at 0.6, and Figure 3c shows the PL intensities of the CdTe/CdSe QDs with different ratios of the MPA stabilizer to the concentration of Cd^2+^. When the [MPA]/[Cd^2+^] ratio ranged from 2.4 to 4.0, the core/shell QDs with high PLQY were obtained in a favorable coating thickness, resulting from an equilibrium coating/dissolution between the QDs and Cd-MPA complexes [17,18]. In the case of the [MPA]/[Cd^2+^] ratio lower than 2.4 or higher than 4.0, it was found that the PL intensities were decreased. The reason is accounted for the effects of the MPA stabilizer on the QD surface or giving rise to non-radiative defects [19]. Thus, the CdTe/CdSe QDs exhibit a relatively good quality when the ratio of [MPA] to [Cd^2+^] is about 1:2.4.

It is known that the CdTe QDs have a great tense to aggregate under the illuminated treatment of a UV lamp [20]. The as-prepared QDs were examined by a UV lamp (16 W) for the comparison of the photostability of CdTe and CdTe/CdSe QDs. The illumination density of all examined solutions was normalized by adjusting their absorption values to 0.2 at the first excitation absorption peak. Figure 3d shows the PL intensity of CdTe and CdTe/CdSe QDs versus the illuminated treatment time. It is shown that the PL intensity of the CdTe QDs quickly decreased under UV treatment and almost faded till 35 min of UV treatment. In contrast, CdTe/CdSe QDs were much more stable against the UV treatment whose PL intensity was enhanced at the initial stage and was held for a long time, 25 min; at 35 min, it just dropped to its original intensity. This improvement of the photostability of CdTe/CdSe QDs was probably due to the protection of the shell to prevent the oxidization of Te^2- ^on the surface of CdTe QDs, thus retained the PL intensity [21]. The enhanced photostability of CdTe/CdSe QDs can be an evidence of the successful formation of core/shell heterostructures.

**Figure 4:**
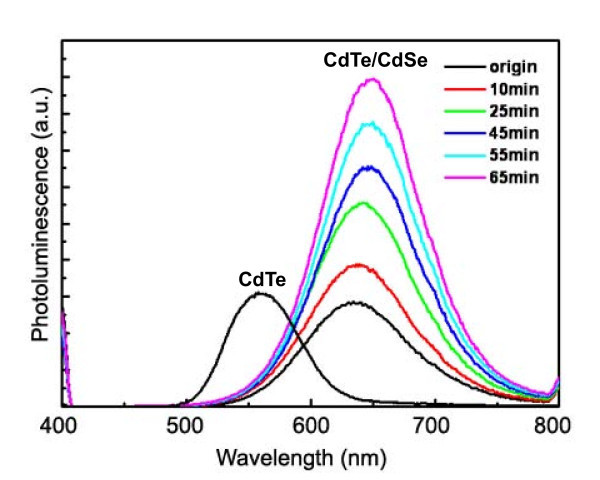
**Results of UV illumination treatment of the as-prepared CdTe/CdSe core/shell QDs.** PL spectra of CdTe/CdSe QDs after UV treatment, which indicates that the PL intensity of the CdTe/CdSe QDs could be enhanced by the lasting time of UV illumination. The PL intensities of the as-prepared CdTe/CdSe QDs were recorded at the same absorbance values at the first excitation the absorption peak.

We also perform the UV-illuminated treatment for improving the PLQY of our as-received CdTe/CdSe QDs. In our case, we found that the adding of 3-MPA to the as-prepared CdTe/CdSe QD solution caused a decrease of the PL intensity. To avoid this kind of fluorescence quenching but still provide S^2- ^for illumination, we made a solution including Cd^2+ ^and MPA (molar ratio [Cd^2+^]/[MPA] = 1:2.4) instead of pure MPA molecule, and the PH value of the solution was adjusted to 11.2. The as-prepared CdTe/CdSe QDs were diluted to the same concentration with the corresponding CdTe QDs and illuminated treatment with UV lamps, and the results are shown in Figure 4. It was found that the PL intensity of the as-prepared CdTe/CdSe QDs was greatly enhanced during illumination. After 65 min of illumination, the PL intensity of CdTe/CdSe achieved at the highest level with the PLQY of 45%. The UV illumination provides a novel photochemical treatment approach for the enhancement of the PLQY of the QDs. Similar results have been reported elsewhere, such as the photoetching of thiol-capped CdTe [22] and the photochemical treatment of ZnSe nanocrystals [23].

## Conclusions

We demonstrated a quick and low-cost approach to synthesize the water-dispersed CdTe/CdSe type II QDs by the microwave-assisted approach. The as-prepared core/shell QDs were water-dispersed and had good crystallinity. The PL performance can be optimized by appropriately adjusting molar ratios of the precursors and the reaction temperature as well as the time of microwave irradiation. The emission wavelength was tunable from 530 to 680 nm by adjusting the reaction time for the increase of shell thickness. Under the UV-illuminated treatment, the PLQY of the core/shell QDs was enhanced to 45% in the presence of Cd^2+ ^and 3-mercaptopropionic acid. The lifetime of the CdTe/CdSe QDs was lasting more than that of the CdTe core QDs, which has the potential applications as sensitizers for solar cells.

## Competing interests

The authors declare that they have no competing interests.

## Authors' contributions

All the authors contributed to writing of the manuscript. LMS carried out the experiments under the instruction of XYK
